# A combined phase I/IIa study of the safety, bronchodilator and bronchoprotective effects of nebulized RPL554, a dual PDE3/4-inhibitor, in healthy subjects and asthmatics

**DOI:** 10.1186/2045-7022-3-S1-O13

**Published:** 2013-05-03

**Authors:** Luigi Franciosi, Zuzana Diamant, Nicoletta Morelli, Marieke de Kam, Adam Cohen, Michael Walker, Clive Page

**Affiliations:** 1 Verona Pharma Plc, Clinical Research, UK; 2 University Medical Centre Groningen, General Practice, the Netherlands; 3 Centre for Human Drug Research (CHDR), Clinical Research, the Netherlands; 4 Centre for Human Drug Research (CHDR), Statistics, the Netherlands; 5 University of British Columbia, Anesthesiology, Pharmacology and Therapeutics, Canada; 6 King’s College London, Sackler Institute of Pulmonary Pharmacology, UK

## Rationale

RPL554 is a novel dual phosphodiesterase (PDE) 3 and 4 isoenzymes inhibitor that has been demonstrated to have both potent, long-acting bronchodilator and anti-inflammatory activity in preclinical models.

## Objectives

To evaluate the safety, bronchodilator and bronchoprotective effects of single ascending doses of RPL554 in healthy subjects and asthmatics.

## Methods

The safety of RPL554 0.003 and 0.009 mg/kg was evaluated in 18 healthy males (age 18-41 years; weight 58-90 kg; height 168-196 cm; BMI 18-29 kg/m^2^) in a randomised, double-blind study. For each dose group, 6 subjects inhaled RPL554 and 3 placebo. Study medication was administered by a calibrated electronic nebuliser and oro-nasal mask. Subsequently, RPL554 0.009 and 0.018 mg/kg were evaluated in 6 asthmatic males (22-29 years; 58-98 kg; 174-198 cm; BMI 19-27 kg/m^2^, FEV1 75-118% pred.) using an open, adaptive design to select an effective dose. This dose (0.018 mg/kg) was further evaluated in a randomised, double-blind, placebo-controlled, cross-over study in 10 allergic asthmatics (20-50 years; 67-112 kg; 171-191 cm; BMI 21-33 kg/m^2^, FEV1 82-112% pred.; baseline PC20(methacholine=MCh) 0.07-1.49 mg/ml; not on controller medication) to assess its bronchodilator effects and bronchoprotective activity against methacholine (MCh) challenge.

## Results

Inhaled RPL554 0.003 to 0.018 mg/kg was well-tolerated in all subjects studied. In the asthmatics, RPL554 0.018 mg/kg produced a rapidly progressive, sustained bronchodilation with a mean maximal increase in FEV1 of 520 ml (95% CI: 320-720 ml; p=0.0061), or 15% increase from baseline. Furthermore, a single dose of RPL554 increased PC20 (MCh) by a mean of 1.46 doubling doses (95% CI: 0.63 **–** 2.28; p=0.004) *versus* placebo. Adverse events were mild, transient and generally of equal frequency in RPL554 and placebo treated groups. No significant changes in heart rate or systemic blood pressure were noted with RPL554 when compared to placebo.

## Conclusions

RPL554 was well-tolerated in the healthy subjects and asthmatics studied. In patients with mild asthma, a rapid, potent and long-lasting bronchodilator response was produced. The maximum bronchodilator effect appeared at least comparable to published values of inhaled salbutamol. RPL554 has the potential to become a first-in-class bronchodilator for airways obstruction.

